# Dietary Lipid Sources and Lysolecithin Modulate Growth, Lipid Metabolism, Hepatopancreatic Health, and Intestinal Microbiota in *Macrobrachium rosenbergii*

**DOI:** 10.1155/anu/8067288

**Published:** 2026-10-27

**Authors:** Zongsheng Qiu, Xinyi Wei, Jianle Yang, Yuqiang Wei, Shasha Liu, Jianhua Zhao, Qiyou Xu, Clement R. de Cruz

**Affiliations:** ^1^ School of Life Sciences Huzhou Normal University Huzhou China; ^2^ International Institute of Aquaculture and Aquatic Sciences Universiti Putra Malaysia Batu 7, Jalan Kemang 6, Teluk Kemang, Si Rusa Port Dickson 71050 Negeri Sembilan Malaysia upm.edu.my; ^3^ Linyi Zhengnengliang Biological Co., Ltd. Linyi China; ^4^ Department of Aquaculture Faculty of Agriculture Universiti Putra Malaysia Serdang 43400 Malaysia upm.edu.my

**Keywords:** hepatopancreatic health, immune response, intestinal microbiota, lipid metabolism, lysolecithin, *Macrobrachium rosenbergii*

## Abstract

This study evaluated the effects of dietary lipid sources and lysolecithin (LL) supplementation on the growth performance, lipid metabolism, hepatopancreatic histology, and intestinal microbiota of *Macrobrachium rosenbergii*. A 56‐day feeding trial was conducted using six isonitrogenous diets containing fish oil (FO), soybean oil (SO), or a 1:1 FO–SO blend (mixed oil [MO]), and each supplemented with or without 0.5% LL. Results indicated a significant interaction between dietary lipid sources and LL supplementation on growth performance (*p* < 0.05). The MO diet supplemented with LL yielded the highest growth rates, while LL supplementation significantly improved feed efficiency (*p* < 0.05). Regarding histological status, the dietary SO treatment induced a significant increase in hepatopancreatic lipid deposition (*p*  < 0.0001) and histological alterations, while LL supplementation reduced lipid deposition and improved hepatopancreatic morphology. Dietary lipid source significantly affected muscle n‐3 polyunsaturated fatty acids (PUFAs; *p* < 0.01). However, a significant interaction between dietary lipid source and LL supplementation was observed specifically for hepatopancreatic total n‐3 PUFA (*p* = 0.0053). Gene expression analysis confirmed that LL modulates lipid metabolism in a source‐dependent manner, upregulating fatty acid oxidation pathways (*Pparα* and *Cpt1*) in FO groups while influencing synthesis pathways (*FAS*) in SO groups (*p* < 0.05). Furthermore, LL supplementation significantly altered the composition of intestinal microbiota, specifically enriching beneficial bacterial genera in dietary SO‐treatment (*p* < 0.05). In conclusion, the dietary treatment combining MOs and LL showed beneficial effects on growth performance, lipid metabolism, and hepatopancreatic health in *M. rosenbergii*.

## 1. Introduction

*Macrobrachium rosenbergii* represents an important form for production in aquaculture using freshwater conditions across regions that show tropical and subtropical features [1, 2]. Data from FAO indicate that production for *M. rosenbergii* reached more than 313,000 tons in 2021, and this production showed a value exceeding 2.45 billion USD [3, 4]. The development of intensive approaches for production requires the development of feed that provide high levels of performance, limit impacts on conditions in the environment, and use resources that show lower relative costs for maintaining development of the industry that can continue over time.

Lipids provide important high‐energy support for the development, molting, and reproduction that occur in crustaceans, and these lipids function as starting materials for important molecules that include essential fatty acids, phospholipids, and steroids [5–7]. Fish oil (FO) has been considered the benchmark lipid source in aquaculture nutrition. This is largely attributed to its high levels of n‐3 long‐chain polyunsaturated fatty acids (PUFAs), especially eicosapentaenoic acid (EPA) and docosahexaenoic acid (DHA), which play essential roles in growth and physiological regulation in aquatic species [8, 9]. However, the stagnation of global FO production and escalating prices, driven by declining wild fishery resources, have severely constrained the cost‐effectiveness and sustainability of the aquaculture industry [10]. Consequently, the evaluation of vegetable oils as viable alternatives has become a major research priority in aquaculture nutrition.

Soybean oil (SO) is widely used to replace FO because of its high yield, low price, and rich linoleic acid (18:2n‐6) [11, 12]. However, the ability of *M. rosenbergii* to synthesize and transform long‐chain n‐3 fatty acids is limited, and the whole SO diet is easy to cause an imbalance of the n‐3/n‐6 ratio [13, 14]. Excessive substitution with SO compromises the growth performance and induces lipid deposition and oxidative stress in the hepatopancreas. These metabolic disorders often provoke inflammatory responses, thereby suppressing immune function and exacerbating tissue damage [15–18]. Developing approaches that increase the use of vegetable oil and reduce negative effects on metabolism remains a main challenge.

The use of functional additives in feed provides a possible approach for improving lipid use in aquatic animals [19–23]. Lysolecithin (LL) is a type of lysophospholipid that develops from natural lecithin when phospholipase A2 (PLA2) removes the fatty acid at the sn‐2 position [24, 25]. LL exhibits a higher hydrophilic–lipophilic balance (HLB), which improves its amphiphilic properties. This facilitates the reduction of oil and water interfacial tension and enhances lipid emulsification and mixed micelle formation. These effects enhance lipid digestion and facilitate the absorption and distribution of lipids from the intestine to target tissues [26, 27]. Previous studies examine pacific white shrimp (*Litopenaeus vannamei*) [28], black tiger shrimp (*Penaeus monodon*) [29], largemouth bass (*Micropterus salmoides*) [29], and rainbow trout (*Oncorhynchus mykiss*) [30]. These structures relate to the transport of lipids and relate to the metabolism of lipids [31–33]. Beyond its role in lipid digestion, LL has been reported to improve the host physiological status by regulating the composition and metabolic activity of the intestinal microbiota [34, 35].

Previous studies have shown that mixed fish and vegetable oils can provide a more balanced fatty acid profile and reduce the reliance on FO in aquafeeds [36, 37]. Meanwhile, dietary LL has been reported to improve growth performance, lipid utilization, antioxidant capacity (AOC), and hepatopancreatic health in *M. rosenbergii* and other crustaceans [28–30]. However, these nutritional strategies have largely been investigated separately. It remains unknown whether the effects of LL depend on the dietary lipid source, particularly when FO and SO are used in combination. Therefore, the interactive effects of lipid source and LL on growth performance, hepatopancreatic lipid metabolism, fatty acid deposition, and intestinal microbiota in *M. rosenbergii* require further investigation.

## 2. Methods and Materials

### 2.1. Animal Ethic

All experimental procedures involving animals were conducted in compliance with the Guidelines for the Care and Use of Laboratory Animals in China and were reviewed and approved by the Animal Ethics Committee of Huzhou University (20241201).

### 2.2. Experimental Diets

Six isonitrogenous and isoenergetic experimental diets were prepared. Lipid sources were FO, SO, and a MO blend (formulated by mixing FO and SO at a 1:1 ratio). The 1:1 FO:SO ratio was selected as a practical 50% FO replacement level, providing an intermediate lipid‐source treatment between the FO and SO diets while maintaining the same total supplemental oil level. This ratio was also intended to combine the n‐3 long‐chain PUFA supplied by FO with the n‐6 PUFA‐rich characteristics of SO, thereby providing a more balanced lipid background for evaluating the effects of LL. Each lipid source was supplemented with or without 0.5% LL, resulting in six experimental groups: FO, FO‐LL, SO, SO‐LL, MO, and MO‐LL. The supplementation level of 0.5% LL was selected based on our previous dose–response study, in which the optimal dietary LL level for *M. rosenbergii* was estimated to be approximately 0.48% based on growth performance [38]. The formulation and proximate composition of the diets are presented in Table 1, and the fatty acid profiles are shown in Table 2. For feed preparation, all dry ingredients were ground, passed through a 60‐mesh sieve, and thoroughly mixed according to the formulation. Subsequently, the respective lipid sources and LL were incorporated, followed by the addition of an appropriate amount of distilled water to form a homogeneous dough. The dough was processed into 1.5‐mm pellets using a pelletizer, dried at 40°C for 12 h, and stored at −20°C until use.

**Table 1:** Proximate composition of the experimental diets (% dry weight basis).

Ingredients (%)	FO	FO‐LL	SO	SO‐LL	MO	MO‐LL
Fish meal	25.00	25.00	25.00	25.00	25.00	25.00
Poultry by‐product	6.00	6.00	6.00	6.00	6.00	6.00
Shrimp meal	5.00	5.00	5.00	5.00	5.00	5.00
Brewers dried yeast	6.00	6.00	6.00	6.00	6.00	6.00
Soybean meal	20.00	20.00	20.00	20.00	20.00	20.00
Starch	15.50	15.50	15.50	15.50	15.50	15.50
Wheat gluten	6.00	6.00	6.00	6.00	6.00	6.00
Cholesterol	0.50	0.50	0.50	0.50	0.50	0.50
Fish oil	4.00	4.00	0.00	0.00	2.00	2.00
Soybean oil	0.00	0.00	4.00	4.00	2.00	2.00
Lysolecithin (LL)^c^	0.00	0.50	0.00	0.50	0.00	0.50
Ca(H_2_PO_4_)_2_	2.00	2.00	2.00	2.00	2.00	2.00
Cellulose	5.50	5.00	5.50	5.00	5.50	5.00
Mineral premix^a^	1.00	1.00	1.00	1.00	1.00	1.00
Vitamin premix^b^	1.00	1.00	1.00	1.00	1.00	1.00
Choline chloride (50%)	0.50	0.50	0.50	0.50	0.50	0.50
Carboxymethyl cellulose	2.00	2.00	2.00	2.00	2.00	2.00
Analyzed nutrients composition (% dry matter basis)						
Crude protein	40.76	41.39	41.48	41.32	40.93	41.84
Crude lipid	8.52	8.45	8.41	8.56	8.43	8.52
Ash	12.99	12.11	12.74	11.69	12.09	10.91

^a, b^The vitamin and mineral premix composition was adapted from our previous study [39].

^c^ Linyi Positive Energy Biotechnology Co., Ltd (Shandong, China).

**Table 2:** Fatty acids composition of experimental diets (% of total fatty acids).

Fatty acid	FO	FO‐LL	SO	SO‐LL	MO	MO‐LL
C14:0	2.78	3.69	2.98	2.70	2.32	2.08
C16:0	24.91	23.03	21.87	21.07	25.39	25.52
C18:0	4.99	4.41	3.88	4.15	5.61	4.88
C16:1	4.35	4.48	4.41	3.67	4.00	3.54
C18:1	17.61	17.31	16.29	16.48	18.04	17.37
C18:2n‐6	19.96	22.64	23.88	29.19	19.48	24.42
C20:4n‐6	2.22	2.08	2.67	1.71	2.82	2.46
C18:3n‐3	1.59	1.93	2.33	3.24	1.64	2.32
C20:5n‐3	6.86	6.64	7.43	6.09	5.81	5.05
C22:6n‐3	10.71	8.72	9.88	7.85	11.39	7.98
∑SFA	32.68	31.13	28.73	27.92	33.32	32.48
∑MUFA	21.95	21.79	20.70	20.14	22.04	20.91
∑n‐6 PUFA	22.17	24.72	26.56	30.90	22.30	26.88
∑n‐3 PUFA	19.16	17.28	19.64	17.18	18.84	15.35

*Note:* Not all analyzed fatty acids are included in this table; only the major ones are presented. Fatty acids present in minor or trace amounts, or those not detected, are omitted. Values represent the average of two measurements.

### 2.3. Prawn and Feeding Trial

*Macrobrachium rosenbergii* were obtained from Zhejiang Guoliang Aquatic Science and Technology Co., Ltd. (Zhejiang, China). The 56‐day feeding trial was conducted in a recirculating aquaculture system at Huzhou University. Prior to the experiment, all prawns were acclimatized to the experimental conditions for 2 weeks. Subsequently, 2400 healthy prawns of uniform size (~0.34 g) were randomly allocated to 24 culture tanks (500 L each), comprising four replicates per treatment with 100 prawns per tank. The 24 tanks were connected to a common recirculating aquaculture system. Each tank received water through a separate inlet and discharged water through an individual outlet into the common filtration system. The tanks were physically separated, and prawns and feed were confined to their respective tanks. Each tank was considered an experimental unit, with four replicate tanks per dietary treatment. During the experiment, prawns were fed twice daily (08:00 and 18:00) under a natural photoperiod. The initial feeding rate was set at 8% of body weight per day and was gradually reduced to 5% as the prawns grew. The daily ration was adjusted weekly according to the estimated biomass and observed feeding response of each tank. During each feeding, feed was supplied gradually until apparent satiation, as indicated by a clear reduction in feeding activity. Little to no uneaten feed remained because the prawns continued to consume residual pellets after feeding. Therefore, uneaten feed was not quantitatively collected. Water quality parameters were maintained stable throughout the trial: water temperature 28.0 ± 1.0°C, dissolved oxygen (DO) approximately 6 mg/L, ammonia nitrogen <0.05 mg/L, and pH 7.5–7.8.

### 2.4. Sample Collection and Preparation

At the end of day‐56 feeding trial, the number of surviving prawns in each tank was counted, and the total weight was recorded. Samples for whole‐body composition analysis were immediately stored at −20°C. Hemolymph was collected from three randomly selected prawns per replicate using 1 mL syringes pre‐loaded with an equal volume of Alsever solution (R1016, Solarbio, China). The samples were kept at 4°C for 24 h and then centrifuged (2000 × *g*, 10 min, 4°C), and the supernatant was collected and stored at −80°C for biochemical and enzyme activity analyses. The remaining prawns were anesthetized on ice and dissected. Tissue samples were collected per dietary group as follows: hepatopancreas and muscle tissues from 12 prawns were stored at −20°C for fatty acid profile analysis, and hepatopancreas samples from three prawns were fixed in 4% paraformaldehyde for histological observation. And hepatopancreas samples from another 12 prawns were stored at −80°C, with six allocated for biochemical analysis and six for gene expression analysis.

### 2.5. Growth Performance

At the end of day‐56 feeding trial, the weight gain (WG), feed conversion ratio (FCR), specific growth rate (SGR), and survival rate (SR) were determined using the specified formulas:

WG %=Final weight−initial weightInitial weight×100,

FCR=Feed intake gweight gain g,

SGR%day−1=Ln final weight g−Ln initial weight gExperimental period (days)×100,

SR% =Final prawn numberInitial prawn number×100.

### 2.6. Proximate and Fatty Acid Analysis

The proximate composition of experimental diets and whole prawn samples from each treatment was analyzed according to standard methods [40]. The moisture content was determined by drying samples to a constant weight using a freeze‐dryer. The crude protein content was determined using a Dumas nitrogen analyzer (DUMATHERM N Pro, Gerhardt, Germany). Crude lipid was extracted using a chloroform–methanol mixture (2:1, *v/v*) and determined gravimetrically, following the method described by Folch et al. [41]. The ash content was determined by incineration in a muffle furnace at 550°C for 8 h. For fatty acid analysis, lipids extracted from diets and tissues were saponified with 0.5 N methanolic NaOH and converted into fatty acid methyl esters (FAMEs). Fatty acid profiles were analyzed using a GC‐2010 Plus gas chromatograph (Shimadzu, Japan), following the protocol described by Qiu et al. [42].

### 2.7. Biochemical Analysis

The contents of plasma total cholesterol (TC), triglyceride (TG), free fatty acid (FFA), glucose (GLU), and hepatopancreatic malondialdehyde (MDA), as well as the activities of total AOC (T‐AOC), superoxide dismutase (SOD), and lipase (LPS), were determined using commercial kits (Nanjing Jiancheng Bioengineering Institute, Nanjing, China). All assays were performed in strict accordance with the manufacturer’s instructions.

### 2.8. Histological Analysis

Hepatopancreas tissues were routinely fixed, dehydrated, and embedded in paraffin, and 3–10 μm paraffin sections were cut using a rotary microtome (Leica). Following standard HE staining steps, sections were dewaxed with xylene, rehydrated with gradient ethanol, stained with hematoxylin, differentiated with acid alcohol, and blued with ammonia water. Subsequently, they were counterstained with eosin, dehydrated with absolute ethanol, cleared in xylene, mounted with neutral resin, and observed under an optical microscope.

For oil red O staining, fresh tissues were fixed with 4% paraformaldehyde and then dehydrated in 15% and 30% sucrose solutions until the tissues sank to the bottom. Frozen sections of 8–10 μm were prepared using a cryostat (Leica 6250) and stored at −20°C for later use. During staining, sections were rewarmed and dried, incubated with oil red O working solution, briefly differentiated with 75% ethanol, counterstained with hematoxylin, blued with ammonia water, and finally mounted with glycerol gelatin.

Lipid deposition was quantified as the relative area (%) of oil red O‐positive lipid droplets, whereas H&E‐stained sections were evaluated descriptively based on hepatopancreatic tubular organization and cytoplasmic vacuolation. No standardized histopathological scoring system was applied.

### 2.9. Gene Expression Analysis

The relative expression levels of *β-actin*, *SREBP*, *Pparα*, *Pparγ*, *FABP*, *CPT1*, *CPT2*, *ACOX1*, *ACC*, *FAS*, *DGAT*, and *Nrf2* were determined by real‐time quantitative PCR (RT‐qPCR).The specific primer sequences used in this study are listed in Table 3. Total RNA extraction was performed using the Trizol method. Briefly, 50 mg of hepatopancreas tissue was homogenized in 1 mL of the Trizol reagent. The homogenate was incubated at room temperature for 5 min, followed by the addition of 200 μL chloroform. The mixture was shaken vigorously for 15 s, incubated for 3 min, and centrifuged at 12,000 rpm for 15 min at 4°C. The upper aqueous phase was collected and mixed with an equal volume of isopropanol. After standing at room temperature for 20 min, the mixture was centrifuged at 12,000 rpm for 10 min at 4°C. The supernatant was discarded, and the pellet was washed with 1 mL of 75% ethanol, centrifuged at 7500 rpm for 5 min at 4°C, air‐dried for 5–10 min, and dissolved in 40 μL of RNase‐free water. RNA concentration and purity were assessed spectrophotometrically prior to cDNA synthesis. RNA samples with an A260/A280 ratio of approximately 1.8–2.1 were considered acceptable for downstream analysis. First‐strand cDNA synthesis was performed using the Evo M‐MLV Reverse Transcription Kit (AG11728, Accurate Biology, Hunan, China). The 20 μL reaction system consisted of 2 μL gDNA Clean Mix, 4 μL 5× RT Reaction Mix, 1 μg total RNA, and RNase‐free water up to 20 μL. The reaction protocol involved incubation at 37°C for 15 min followed by 85°C for 5 s. The resulting cDNA was stored at −20°C. qPCR amplification was carried out using the SYBR Green Pro Taq HS Kit (AG11701). The 20 μL reaction mixture contained 10 μL 2× Premix, 0.4 μL of each forward and reverse primer (10 μmol/L), 2 μL cDNA, and 7.2 μL RNase‐free water. The thermal cycling conditions were: pre‐denaturation at 95°C for 30 s, followed by 40 cycles of denaturation at 95°C for 5 s and annealing/extension at 60°C for 30 s. Melting curve analysis was performed to verify amplification specificity. The amplification efficiencies of all primer pairs ranged from 98% to 101%. Relative gene expression levels were calculated using the 2^−ΔΔCT^ method.

**Table 3:** Primer sequences used for gene expression analysis in *M. rosenbergii*.

Genes	Primer sequences (5′–3′)	GeneBank and reference
*β-actin*	F: AGAGAGACGGTCGGTGTTTG	NC_089772.1
	R: TACAACCAACGCGGCTACTT	
*SREBP*	F: CTCCGATTGGCCGAAAGACT	NC_089743.1
	R: CTCCTCACAAAACCCCTCCC	
*Pparα*	F: ATGTCCAGTCCTCCTGTACTCC	[43]
	R: AAGAGCAGAGCCGCAATCAG	
*Pparγ*	F: GAAGGGCACGGCTAAACAAC	NC_089741.1
	R: CAGGTCCACAGGAACACACG	
*FABP*	F: TAGTTCGTCTGGCTTCGTCG	NC_089756.1
	R: GCGACTTGCTCACGACTACT	
*CPT1*	F: TGATGGATGATGAAGCCTAT	[43]
	R: TCCTCCCACCAATCTGTT	
*CPT2*	F: GCTGGATGGACTAAGCGCA	NC_089771.1
	R: GTCTTCTGCTTTGTAACGGCG	
*ACOX1*	F: GGCCTTCAAACAACAGCCAC	NC_089765.1
	R: CTGAGCCATAACCACAGCGT	
*ACC*	F: GGCCAAATCAACGGAGGGTA	NC_089787.1
	R: TTCGCATACACTTGACCGCA	
*FAS*	F: GAGGAATGTACCCGTCCAGC	NC_089790.1
	R: CCAAATCCTCCAAATCAGCCA	
*DGAT*	F: TCACAACCCGACAGACCG	[43]
	R: TGGCGTTTGGAAATAGTAGC	
*Nrf2*	F: CTGGACGAGGAGACTGGA	[43]
	R: ATCTGTGGTAGGTGGAAC	

*Note:* β‐actin was used as the internal reference gene.

Abbreviations: *ACC*, acetyl‐CoA carboxylase; *ACOX1*, acyl‐CoA oxidase 1; *CPT1*, carnitine palmitoyltransferase 1; *CPT2*, carnitine palmitoyltransferase 2; *DGAT*, diacylglycerol O‐acyltransferase; F, forward primer; *FABP*, fatty acid‐binding protein; *FAS*, fatty acid synthase; *Nrf2*, nuclear factor erythroid 2‐related factor 2; *Pparα*, peroxisome proliferator‐activated receptor alpha; *Pparγ*, peroxisome proliferator‐activated receptor gamma; R, reverse primer; *SREBP*, sterol regulatory element‐binding protein.

### 2.10. Analysis of Intestinal Microbiota

Intestinal tissue samples were sent to Shanghai Majorbio Bio‐pharm Technology Co., Ltd. (Shanghai, China) for high‐throughput sequencing of the 16S rRNA gene. Total genomic DNA of the microbial community was extracted using the E.Z.N.A. Soil DNA Kit (Omega Bio‐tek). DNA integrity and concentration were assessed by agarose gel electrophoresis and a NanoDrop 2000 spectrophotometer (Thermo Scientific). Subsequently, the V3–V4 hypervariable region of the 16S rRNA gene was amplified using primers 338F (5′‐ACTCCTACGGGAGGCAGCAG‐3′) and 806R (5′‐GGACTACHVGGGTWTCTAAT‐3′) to construct the sequencing library. Finally, sequencing was performed on the Illumina NextSeq 2000 platform.

### 2.11. Statistical Analysis

All data are expressed as mean ± standard error (S.E.). Prior to analysis, data were tested for normality and homogeneity of variances using the Shapiro–Wilk and Levene’s tests, respectively. Two‐way analysis of variance (ANOVA) was performed to examine the main effects of dietary lipid sources, LL supplementation, and their interactions on growth performance, tissue composition, biochemical parameters, and gene expression. When significant differences were detected (*p* < 0.05), means were compared using Tukey’s multiple range test. Additionally, pairwise comparisons for differentially abundant microbial genera were analyzed using the Wilcoxon rank‐sum test. Statistical analyses were conducted using JMP Pro, Version 16 (SAS Institute Inc., Cary, NC, USA). Graphs were generated using GraphPad Prism 10.0 (GraphPad Software, San Diego, CA, USA).

## 3. Results

### 3.1. Growth Performance

The growth performance, feed utilization, and SR of *M. rosenbergii* are presented in Table 4. The study shows significant interaction patterns between dietary lipid sources and LL levels for FBW, WG, and SGR (*p* < 0.05). Analysis indicated that the MOLL group exhibited FBW, WG, and SGR values that differ significantly from the FO, SO, SOLL, and MO groups (*p* < 0.05). Results show that this group presents higher measures across these factors. Regarding main effects, lipid sources significantly influenced FBW, WG, and SGR (*p* < 0.05), with the MO treatments yielding the highest growth performance and lowest FCR. Similarly, LL levels significantly affected FBW, WG, SGR, and FCR (*p* < 0.05), prawns supplemented with 0.5% LL showed higher FBW, WG, and SGR, and lower FCR compared to the nonsupplemented groups (0%). No significant effects of lipid sources, LL levels, or their interaction were observed on SR (*p* > 0.05).

**Table 4:** Effects of dietary lipid sources and lysolecithin supplementation on growth performance, feed utilization, and survival of *M. rosenbergii*.

Experimental diets		FBW (g)	WG (%)	SGR (%/day)	FCR	SR (%)
Lipid sources	Lysolecithin levels (%)					
FO	0	3.33^b^	852.63^b^	4.02^b^	1.89	69.50
FO	0.5	3.43^ab^	880.88^ab^	4.08^ab^	1.66	71.75
SO	0	3.22^b^	820.57^b^	3.96^b^	1.65	76.00
SO	0.5	3.30^b^	842.22^b^	4.01^b^	1.67	75.25
MO	0	3.28^b^	838.45^b^	4.00^b^	1.65	77.75
MO	0.5	3.65^a^	944.18^a^	4.19^a^	1.53	75.75

Pooled s.e.		0.054	0.156	0.029	0.06	3.540

Main effects						
Lipid sources						
FO		3.39^ab^	866.76^ab^	4.05^ab^	1.77^a^	70.63
SO		3.26^b^	831.40^b^	3.98^b^	1.66^ab^	75.63
MO		3.47^a^	891.32^a^	4.09^a^	1.59^b^	76.75

Lysolecithin levels						
0		3.28^b^	837.22^b^	3.99^b^	1.73^a^	74.42
0.5		3.46^a^	889.09^a^	4.09^a^	1.62^b^	74.25

ANOVA, Pr > *F*						

Lipid sources		0.0043	0.0044	0.0043	0.0228	0.2119
Lysolecithin levels		0.0007	0.0007	0.0007	0.0392	0.9547
Lipid sources × lysolecithin levels		0.0242	0.0265	0.0396	0.1464	0.8286

*Note:* Values are means of four replicates. Mean values in the same column with different superscript letters are significantly different (*p* < 0.05).

Abbreviations: FBW, final body weight; FCR, feed conversion ratio; SGR, specific growth rate; SR, survival rate; WG, weight gain.

### 3.2. Proximate Composition of Whole Prawn

The whole‐body proximate composition of *M. rosenbergii* is presented in Table 5. Lipid sources, LL supplementation levels, and their interaction exerted no significant effects on moisture, crude protein, and ash contents (*p* > 0.05). However, whole‐body crude lipid content was significantly influenced by the main effects of lipid sources (*p* = 0.0009) and LL levels (*p* = 0.0231), while their interaction was not significant (*p* = 0.2677). Regarding LL, prawns supplemented with 0.5% LL exhibited significantly lower whole‐body crude lipid content compared to the nonsupplemented group (0%).

**Table 5:** Effects of dietary lipid sources and lysolecithin supplementation on whole‐body proximate composition of *M. rosenbergii*.

Experimental diets		Moisture (%)	Crude protein (%)	Crude lipid (%)	Ash (%)
Lipid sources	Lysolecithin levels (%)				
FO	0	73.10	16.96	2.52	4.41
FO	0.5	73.32	17.57	2.24	4.56
SO	0	70.64	18.96	2.98	4.65
SO	0.5	74.15	16.77	2.47	3.90
MO	0	73.70	16.91	3.03	4.58
MO	0.5	74.29	15.82	2.99	4.27

Pooled s.e.		1.06	0.71	0.14	0.92

FO		73.21	17.27	2.38^b^	4.48
SO		72.39	17.87	2.72^ab^	4.22
MO		73.99	16.37	3.01^a^	4.43

0		72.48	17.61	2.84^a^	4.51
0.5		73.92	16.72	2.57^b^	4.24

ANOVA, Pr > *F*					

Lipid sources		0.3445	0.1325	0.0009	0.5019
Lysolecithin levels		0.1153	0.1404	0.0231	0.1785
Lipid sources × lysolecithin levels		0.2649	0.1673	0.2677	0.2540

*Note:* Values are means of four replicates. Mean values in the same column with different superscript lowercase letters are significantly different (*p* < 0.05).

### 3.3. Fatty Acid Profiles of Muscle and Hepatopancreas

The fatty acid profiles of the hepatopancreas are presented in Table 6. Lipid sources exerted significant main effects on total saturated fatty acids (ΣSFA), monounsaturated fatty acids (ΣMUFA), n‐3 PUFAs (Σn‐3 PUFA), n‐6 PUFA (Σn‐6 PUFA), and most individual fatty acids (*p* < 0.001). Specifically, the contents of Σn‐3 PUFA in the FO and FOLL groups were significantly higher than those in the SO, SOLL, MO, and MOLL groups (*p* < 0.05). The Σn‐6 PUFA content was highest in the SO treatments, followed by the MO treatments, and lowest in the FO treatments (*p* < 0.05), which was primarily driven by variations in C18:2n6. Additionally, the FO treatments exhibited the highest ΣSFA and ΣMUFA contents. Specifically, ΣSFA levels were significantly higher than in the SO group, whereas ΣMUFA levels were significantly higher than in the MO group (*p* < 0.05). In contrast, the main effects of LL levels were limited, significantly affecting only C16:1 (*p* = 0.0324) without altering major fatty acid categories (*p* > 0.05). However, significant interactions between lipid sources and LL levels were observed for ΣSFA (*p* = 0.0104) and Σn‐3 PUFA (*p* = 0.0053). Further analysis of the interaction revealed that for Σn‐3 PUFA, the MOLL group was higher than the MO group, whereas the FOLL and SOLL groups were lower than their respective controls. Conversely, for ΣSFA, the MOLL group was lower than the MO group, while the FOLL and SOLL groups were higher than the FO and SO groups.

**Table 6:** Effects of dietary lipid sources and lysolecithin supplementation on hepatopancreas fatty acid profiles of *M. rosenbergii*.

Experimental diets		Fatty acids													
Lipid sources	Lysolecithin levels (%)	C14:0	C16:0	C18:0	C16:1	C18:1	C18:3n3	C20:5n3	C22:6n3	C18:2n6	C20:4n6	ΣSFA	ΣMUFA	Σn‐3 PUFA	Σn‐6 PUFA
FO	0	6.96	23.27^ab^	7.73	5.41	23.60	0.88	5.94	6.20	11.32	1.18	37.95^a^	29.01	13.02^a^	12.49
FO	0.5	6.31	24.98^a^	7.89	5.02	22.97	0.93	5.40	5.66	12.16	1.15	39.18^a^	28.00	11.98^ab^	13.31
SO	0	3.01	18.74^e^	8.25	2.18	25.12	2.00	3.93	3.42	26.80	0.70	30.00^c^	27.30	9.35^bcd^	27.50
SO	0.5	3.53	19.40^de^	8.08	2.26	26.36	1.79	2.86	2.59	26.25	0.52	31.01^bc^	28.62	7.24^d^	26.77
MO	0	4.97	22.42^bc^	9.45	3.79	23.05	1.27	4.62	4.25	18.21	1.07	36.84^a^	26.84	7.35^cd^	19.28
MO	0.5	4.85	20.87^cd^	7.69	3.38	23.87	1.59	4.36	4.35	21.84	0.86	33.41^b^	27.24	10.29^abc^	22.70

Pooled s.e.		0.22	0.44	0.48	0.12	0.46	0.13	0.39	0.33	0.98	0.11	0.71	0.52	0.64	0.95

FO		6.64^a^	24.12^a^	7.92	5.22^a^	23.29^b^	1.89^a^	5.67^a^	5.93^a^	11.74^c^	1.16^a^	38.56^a^	28.50^a^	12.50^a^	12.90^c^
SO		4.91^b^	19.07^c^	8.16	2.22^c^	25.74^a^	0.90^c^	3.39^c^	3.00^c^	26.52^a^	0.61^b^	30.50^c^	27.96^ab^	8.29^b^	27.13^a^
MO		3.27^c^	21.64^b^	8.57	3.58^b^	23.46^b^	1.43^b^	4.49^b^	4.30^b^	20.02^b^	0.97^a^	35.12^b^	27.04^b^	8.87^b^	20.99^b^

0		4.98	21.48	8.47	3.79^a^	23.92	1.38	4.83	4.62	18.78	0.98	34.93	27.95	9.94	19.76
0.5		4.89	21.75	7.89	3.55^b^	24.40	1.43	4.21	4.20	20.08	0.84	34.53	27.72	9.83	20.93

ANOVA, Pr > *F*															

Lipid sources		<0.0001	<0.0001	0.3176	<0.0001	0.0003	<0.0001	0.0003	<0.0001	<0.0001	0.0013	<0.0001	0.0440	<0.0001	<0.0001
Lysolecithin levels		0.6488	0.4643	0.1590	0.0324	0.2310	0.6620	0.0752	0.1448	0.1293	0.1552	0.5064	0.5858	0.8437	0.1555
Lipid sources × lysolecithin levels		0.0576	0.0088	0.1421	0.1162	0.1501	0.1820	0.5901	0.3866	0.1382	0.7214	0.0104	0.1155	0.0053	0.1259

*Note:* Values are means of four replicates. Mean values in the same column with different superscript letters are significantly different (*p* < 0.05). Not all analyzed fatty acids are included in this table; only the major ones are presented. Fatty acids present in minor or trace amounts, or those not detected, are omitted.

The fatty acid profiles of the muscle tissue are presented in Table 7. Dietary lipid sources exerted significant main effects on ΣSFA, ΣMUFA, Σn‐3 PUFA, and Σn‐6 PUFA (*p* < 0.001). Specifically, the content of Σn‐3 PUFA in the FO group was significantly higher than that in the SO and MO groups. The content of Σn‐6 PUFA was the highest in the SO group, followed by the MO group and the FO group. Additionally, the contents of ΣSFA and ΣMUFA in the FO group were significantly higher than those in the SO group (*p* < 0.05). Regarding LL, significant main effects were observed for ΣMUFA (*p* = 0.0002) and Σn‐6 PUFA (*p* = 0.0061). The contents of ΣMUFA and Σn‐6 PUFA in the group supplemented with 0.5% LL were significantly lower than those in the group without LL (0%), whereas LL addition had no significant effect on ΣSFA and Σn‐3 PUFA (*p* > 0.05). Furthermore, no significant interactions between lipid sources and LL levels were observed for ΣSFA, ΣMUFA, Σn‐3 PUFA, and Σn‐6 PUFA in muscle (*p* > 0.05).

**Table 7:** Effects of dietary lipid sources and lysolecithin supplementation on muscle tissue fatty acid profiles of *M. rosenbergii*.

Experimental diets		Fatty acids													
Lipid sources	Lysolecithin levels (%)	C14:0	C16:0	C18:0	C16:1	C18:1	C18:3n3	C20:5n3	C22:6n3	C18:2n6	C20:4n6	ΣSFA	ΣMUFA	Σn‐3 PUFA	Σn‐6 PUFA
FO	0	2.33	22.82	6.69	2.57	19.47	0.99^c^	17.67^a^	8.37	11.47	2.83	31.83	22.05	14.31	27.03
FO	0.5	1.83	20.28	6.28	2.11	17.98	0.94^c^	15.18^ab^	7.26	10.95	2.72	28.39	20.08	13.66	23.38
SO	0	1.49	19.54	6.34	1.50	17.24	1.73^a^	13.41^b^	5.35	20.73	1.79	27.38	18.73	22.52	20.49
SO	0.5	0.98	19.26	6.70	0.85	15.71	1.32^b^	14.33^b^	5.23	18.16	2.17	26.94	16.56	20.33	20.88
MO	0	1.19	20.75	7.14	1.31	17.64	1.03^bc^	14.83^ab^	6.43	14.91	2.71	29.09	18.95	17.62	22.29
MO	0.5	1.08	19.86	7.07	1.12	17.15	0.92^c^	13.89^b^	4.27	13.54	2.76	28.00	18.27	16.31	19.08

Pooled s.e.		0.12	0.84	0.43	0.12	0.36	0.06	0.60	0.89	0.55	0.17	1.21	0.38	1.26	0.51

FO		2.08^a^	21.55	6.49	2.34	18.73^a^	0.96^b^	16.43^a^	7.82^a^	11.21^c^	2.78^a^	30.11	21.07^a^	25.20^a^	13.99^c^
SO		1.24^b^	19.40	6.52	1.17	16.47^b^	1.53^a^	13.87^b^	5.29^b^	19.44^a^	1.98^b^	27.16	17.65^b^	20.69^b^	21.42^a^
MO		1.14^b^	20.31	7.10	1.22	17.39^b^	0.97^b^	14.36^b^	5.35^b^	14.23^c^	2.74^a^	28.55	18.61^b^	20.68^b^	16.96^b^

0		1.67^a^	21.04	6.73	1.79^a^	18.12^a^	1.25^a^	15.30	6.72	15.70^a^	2.44	29.43	19.91	23.27	18.15^a^
0.5		1.29^b^	19.80	6.68	1.36^b^	16.94^b^	1.06^b^	14.46	5.59	14.22^b^	2.55	27.78	18.30	21.11	16.77^b^

ANOVA, Pr > *F*															

Lipid sources		<0.0001	0.0706	0.3067	<0.0001	0.0001	<0.0001	0.0026	0.0233	<0.0001	0.0006	0.0878	<0.0001	0.0050	<0.0001
Lysolecithin levels		0.0023	0.0944	0.9063	0.0008	0.0016	0.0034	0.1132	0.1459	0.0064	04339	0.1182	0.0002	0.0583	0.0061
Lipid sources × lysolecithin levels		0.1994	0.4074	0.6775	0.1970	0.2899	0.0309	0.0455	0.5374	0.2210	0.3443	0.4489	0.1450	0.2538	0.3481

*Note:* Values are means of four replicates. Mean values in the same column with different superscript letters are significantly different (*p* < 0.05). Not all analyzed fatty acids are included in this table; only the major ones are presented. Fatty acids present in minor or trace amounts, or those not detected, are omitted.

### 3.4. Plasma Biochemical Parameters

The plasma biochemical parameters of *M. rosenbergii* are presented in Figure 1. Lipid sources, LL levels, and their interaction exerted no significant effects on plasma GLU and FFA concentrations (*p* > 0.05). Regarding plasma TC, significant main effects were observed for lipid sources (*p* = 0.0003) and LL levels (*p* = 0.0033), whereas the interaction was not significant (*p* = 0.0507). Main effects indicated that the TC content in the MO treatments was higher than that in the FO and SO groups, and supplementation with 0.5% LL significantly reduced the overall plasma TC level. Pairwise comparisons revealed that LL supplementation significantly reduced TC levels in the FO (*p* < 0.001) and SO groups (*p* < 0.0001), whereas no significant change was observed in the MO group (*p* > 0.05). For plasma TG, lipid sources (*p* = 0.0141) and LL levels (*p* = 0.0033) exerted significant main effects, and a significant interaction was detected (*p* = 0.0495). Decomposition of the interaction revealed that at 0% LL, the SO group exhibited the highest TG level. However, supplementation with 0.5% LL significantly reduced TG content in the SO group (*p* < 0.001), leading to levels lower than those of the FO and MO groups at the same LL level. In contrast, TG content in the MO group did not significantly decrease with LL supplementation, and consequently, the MO‐LL group exhibited the highest TG levels among all treatments.

**Figure 1:**
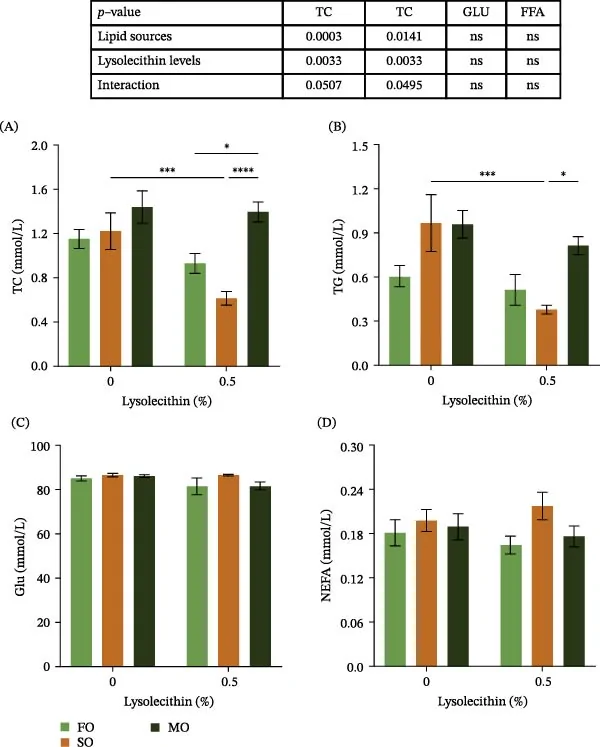
Effects of dietary lipid sources and lysolecithin supplementation on plasma biochemical parameters of *M. rosenbergii*. (A) TC: total cholesterol, (B) TG: triglyceride, (C) GLU: glucose, (D) FFA: free fatty acid. Values are presented as mean ± S.E. (*n* = 6). Asterisks indicate significant differences ( ^∗^ *p* < 0.05,  ^∗∗^ *p* < 0.01,  ^∗∗∗^ *p* < 0.001,  ^∗∗∗∗^ *p* < 0.0001).

### 3.5. Hepatopancreatic AOC and LPS Activity

The hepatopancreatic antioxidant capacities and LPS activity are presented in Figure 2. Lipid sources exerted significant main effects on T‐AOC (*p* = 0.008), SOD (*p* < 0.0001), and LPS (*p* = 0.0003). SOD activity was highest in the FO group, followed by the MO group, and was lowest in the SO group, with significant differences observed among all three groups (*p* < 0.05). LL supplementation exerted significant main effects on SOD (*p* < 0.0001) and MDA (*p* = 0.0009). Irrespective of lipid sources, supplementation with 0.5% LL significantly increased hepatopancreatic SOD activity and decreased MDA content. LL supplementation had no significant effect on T‐AOC and LPS activities (*p* > 0.05). Furthermore, no significant interactions between lipid sources and LL levels were observed for T‐AOC, SOD, MDA, or LPS (*p* > 0.05).

**Figure 2:**
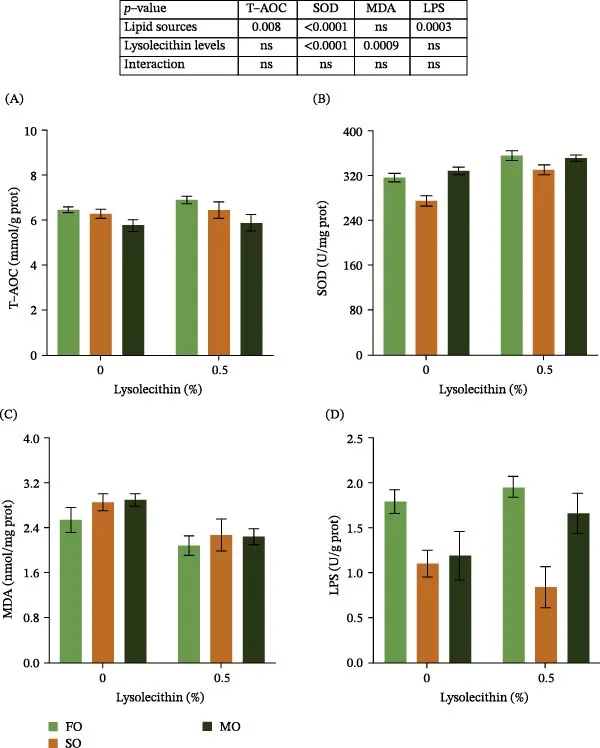
Effects of dietary lipid sources and lysolecithin supplementation on hepatopancreatic antioxidant capacity and lipase activity of *M. rosenbergii*. (A) T‐AOC: total antioxidant capacity, (B) SOD: superoxide dismutase, (C) MDA: malondialdehyde, and (D) LPS: lipase. Values are presented as mean ± S.E. (*n* = 6). Asterisks indicate significant differences ( ^∗^ *p* < 0.05,  ^∗∗^ *p* < 0.01,  ^∗∗∗^ *p* < 0.001,  ^∗∗∗∗^ *p* < 0.0001).

### 3.6. Histological Analysis of Hepatopancreas

Histological observations of the hepatopancreas via HE staining are presented in Figure 3. Each treatment group exhibited typical hepatic tubular structures, and columnar epithelial nuclei were mostly located at the base or center of cells. Different lipid sources induced distinct morphological differences: the hepatic tubules in the FO and MO groups were arranged regularly, and the cytoplasm appeared uniform without obvious vacuolization. However, in the SO group, the arrangement of some hepatic tubules was slightly loose, and slight vacuolar degeneration was observed in the cytoplasm. Upon LL supplementation, the histological integrity of each group was improved compared to the nonsupplemented groups. Specifically, cytoplasmic vacuolation in the SO‐LL group was significantly reduced, and the hepatic tubular structure in the MO‐LL group was the most compact and regular, with no obvious pathological abnormalities observed. Overall, LL supplementation improved the histomorphology of the hepatopancreas, regardless of the lipid source background.

**Figure 3:**
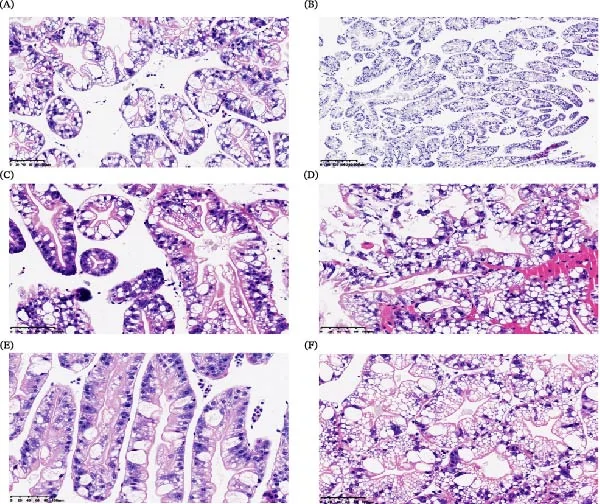
Histological observation of the hepatopancreas of *M. rosenbergii* fed experimental diets (H&E staining). (A) FO, (B) FO‐LL, (C) SO, (D) SO‐LL, (E) MO, and (F) MO‐LL.

The results of oil red O staining of the hepatopancreas and quantitative analysis of the relative area (%) of lipid droplets are presented in Figures 4 and 5. Two‐way ANOVA indicated that lipid sources (*p* < 0.0001), LL levels (*p* = 0.0002), and their interaction (*p* < 0.0001) significantly affected lipid deposition in the hepatopancreas. In the absence of LL (0%), lipid deposition in the SO group was the most severe (relative area 48%), being significantly higher than that in all other groups (*p* < 0.0001). The FO group (20%) ranked second, exhibiting significantly higher levels than the MO group (6%) (*p* < 0.05). Interaction analysis revealed that the addition of 0.5% LL significantly alleviated lipid deposition in the FO and SO groups (*p* < 0.05). In contrast, lipid deposition in the MO‐LL group did not significantly decrease but rather showed a slight increase compared with the MO group. In conclusion, the SO group showed the most severe lipid accumulation. While LL supplementation significantly reduced hepatopancreatic lipid deposition induced by SO and FO, it did not exert the same lipid‐lowering effect in the MO group.

**Figure 4:**
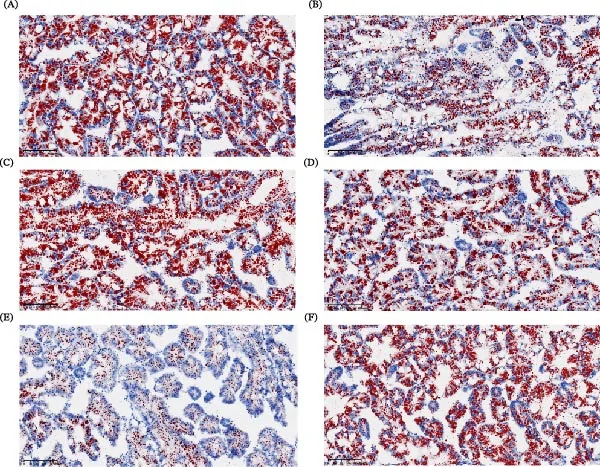
Histological observation of lipid accumulation in the hepatopancreas of *M. rosenbergii* (oil red O staining). (A) FO, (B) FO‐LL, (C) SO, (D) SO‐LL, (E) MO, and (F) MO‐LL.

**Figure 5:**
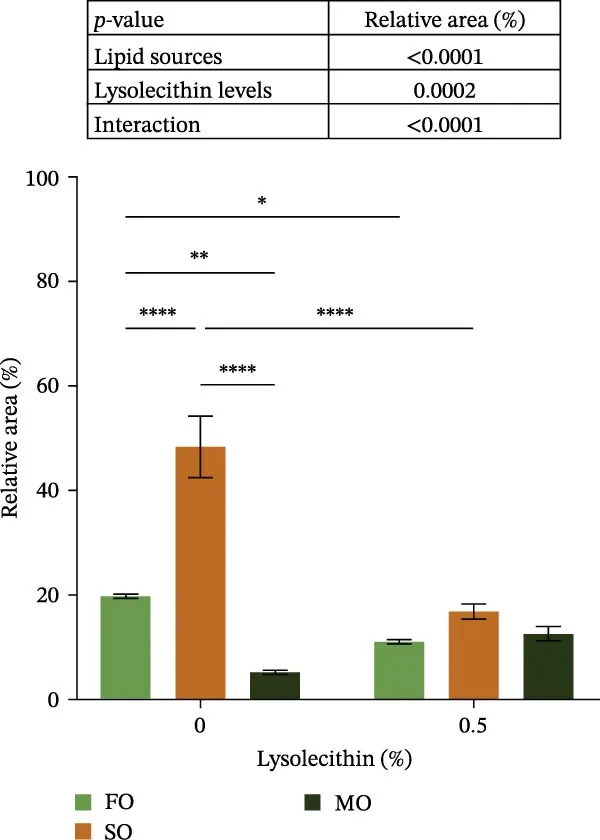
Quantitative analysis of the relative area of lipid droplets in the hepatopancreas of *M. rosenbergii* fed experimental diets. Values are presented as mean ± S.E. (*n* = 4). Asterisks indicate significant differences ( ^∗^ *p* < 0.05,  ^∗∗^ *p* < 0.01,  ^∗∗∗^ *p* < 0.001,  ^∗∗∗∗^ *p* < 0.0001).

### 3.7. Expression of Genes Related to Lipid Metabolism and AOC in Hepatopancreas

Lipid sources and LL supplementation together affected patterns that show the expression of factors relating to lipid use, fatty acid movement and breakdown, fatty acid formation, and response to damage in the hepatopancreas, and the interaction between these treatments produced significant differences for main factors (Figures 6–8). The study examined factors controlling lipid use. Expression of *SREBP* showed significant interaction effects (*p* = 0.0456, Figure 6A). The level in the MO group was high in the absence of LL, while supplementation with LL reduced the expression in the FO group specifically. Expression of *PParα* revealed strong interaction with LL supplementation (*p* < 0.0001, Figure 6B). Expression levels were low across groups that did not include LL; however, supplementation with 0.5% LL increased *PParα* in the FO group, producing the highest values among treatment groups. Expression of *PPar* was primarily driven by the main effects of lipid sources (*p* < 0.0001) and LL (*p* = 0.0057), with no significant interaction observed (*p* > 0.05, Figure 6C). The overall expression level of the FO group was higher than that of the SO group and MO group, while LL supplementation produced an overall decrease in expression. Factors relating to fatty acid transport and oxidation (*FABP*, *CPT1*, and *ACOX1*) showed a consistent pattern that indicated significant interaction between lipid source and LL (*p* < 0.01, Figure 7A,B,D). LL supplementation increased the expression of these factors. This effect was most clear in the FO group. The expression of *FABP*, *CPT1*, and *ACOX1* in the FO‐LL group was higher than that in all other groups. For *CPT2*, the MO group showed high expression at the initial level, and LL supplementation increased the expression in the FO group (Figure 7C). For processes relating to synthesis and storage of fatty acids, the expression of *ACC* and *DGAT* was primarily driven by lipid sources (*p* < 0.01, Figure 8A,C), with the SO group exhibiting the highest overall expression levels. No significant effects of LL supplementation or interaction were observed for these two genes (*p* > 0.05). However, a significant interaction was detected for FAS expression (*p* = 0.0004, Figure 8B). Specifically, LL supplementation significantly upregulated FAS expression in the SO group, suggesting that LL may further promote de novo fatty acid synthesis in treatments using SO. Regarding antioxidant regulation, *Nrf2* expression showed a significant interaction (*p* < 0.0001, Figure 8D). Consistent with other oxidation‐related genes, LL supplementation substantially increased *Nrf2* expression in the FO group, approximately three‐fold compared to the nonsupplemented group, while the SO and MO groups showed limited responses.

**Figure 6:**
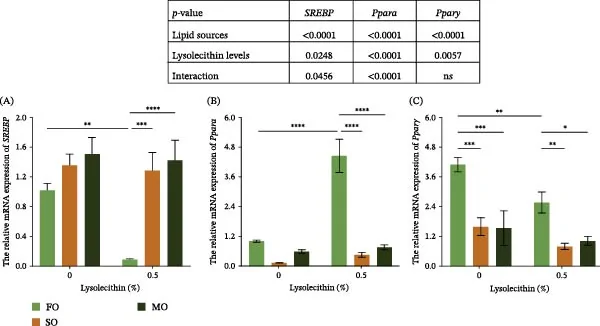
Effects of dietary lipid sources and lysolecithin supplementation on the relative expression of genes related to lipid metabolism regulation in the hepatopancreas of *M. rosenbergii*. (A) *SREBP*: sterol regulatory element‐binding protein, (B) *Pparα*: peroxisome proliferator‐activated receptor alpha, and (C) *Pparγ*: peroxisome proliferator‐activated receptor gamma. Values are presented as mean ± S.E. (*n* = 6). Asterisks indicate significant differences ( ^∗^ *p* < 0.05,  ^∗∗^ *p* < 0.01,  ^∗∗∗^ *p* < 0.001,  ^∗∗∗∗^ *p* < 0.0001).

**Figure 7:**
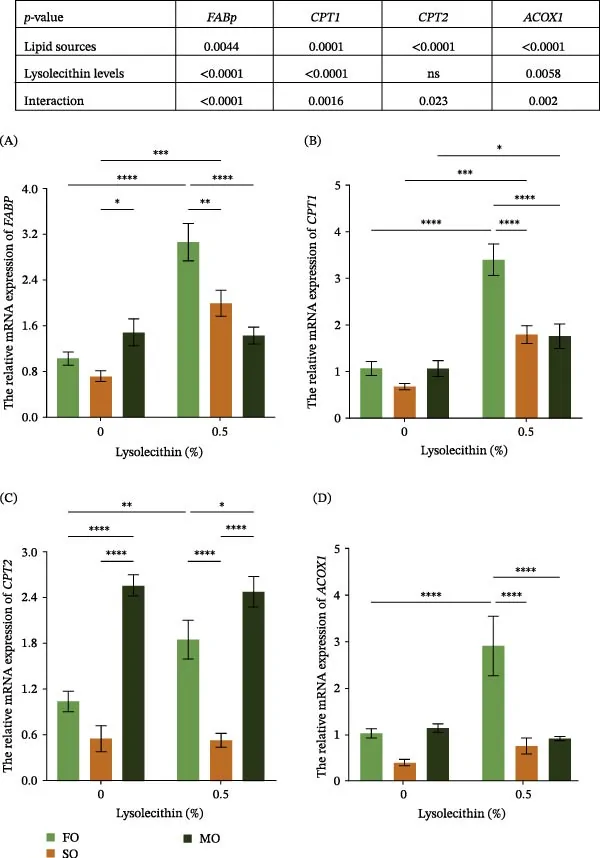
Effects of dietary lipid sources and lysolecithin supplementation on the relative expression of genes related to fatty acid transport and oxidation in the hepatopancreas of *M. rosenbergii*. (A) *FABP*: fatty acid‐binding protein, (B) *CPT1*: carnitine palmitoyltransferase 1, (C) *CPT2*: carnitine palmitoyltransferase 2, and (D) *ACOX1*: acyl‐CoA oxidase 1. Values are presented as mean ± S.E. (*n* = 6). Asterisks indicate significant differences ( ^∗^ *p* < 0.05,  ^∗∗^ *p*  < 0.01,  ^∗∗∗^ *p* < 0.001,  ^∗∗∗∗^ *p* < 0.0001).

**Figure 8:**
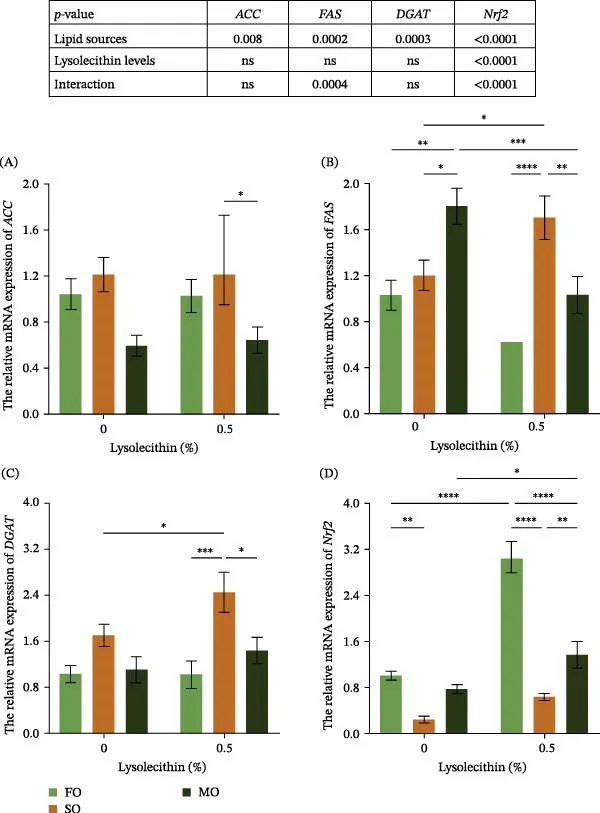
Effects of dietary lipid sources and lysolecithin supplementation on the relative expression of genes related to lipid synthesis and antioxidant capacity in the hepatopancreas of *M. rosenbergii*. (A) *ACC*: acetyl‐CoA carboxylase, (B) *FAS*: fatty acid synthase, (C) *DGAT*: diacylglycerol O‐acyltransferase, and (D) *Nrf2*: nuclear factor erythroid 2‐related factor 2. Values are presented as mean ± S.E. (*n* = 6). Asterisks indicate significant differences ( ^∗^ *p* < 0.05,  ^∗∗^ *p* < 0.01,  ^∗∗∗^ *p* < 0.001,  ^∗∗∗∗^ *p* < 0.0001).

### 3.8. Intestinal Microbiota

#### 3.8.1. Alpha Diversity of Intestinal Microbiota

Based on 16S rRNA gene high‐throughput sequencing, the alpha diversity indices of the intestinal microbiota in *M. rosenbergii* were evaluated (Table 8). Overall, two‐way ANOVA indicated that the main effects of lipid sources and LL supplementation on Shannon, Simpson, Ace, and Chao indices were not significant. Regarding the Shannon index, although numerical fluctuations were observed, statistical analysis revealed no significant differences among groups, nor was there a significant interaction between lipid sources and LL supplementation. Simpson index analysis revealed a marginally significant interaction effect between lipid sources and LL supplementation. Post hoc tests indicated that LL supplementation significantly increased the Simpson index in the SO group, suggesting a significant increase in community dominance, whereas the FO and MO groups remained relatively stable. Furthermore, no significant differences were observed among experimental groups for the Ace and Chao indices, which reflect community richness.

**Table 8:** Effects of dietary lipid sources and lysolecithin supplementation on alpha diversity indices of intestinal microbiota of *M. rosenbergii*.

Experimental diets		Shannon	Simpson	Ace	Chao
Lipid sources	Lysolecithin levels (%)				
FO	0	3.59	0.10^b^	335.66	335.81
FO	0.5	3.73	0.09^b^	311.81	311.16
SO	0	3.63	0.11^b^	337.38	337.05
SO	0.5	2.60	0.31^a^	272.92	272.77
MO	0	3.27	0.16^ab^	285.35	285.02
MO	0.5	3.25	0.18^ab^	302.26	302.62

Pooled s.e.		0.29	0.05	28.73	28.79

FO		3.66	0.10	323.73	323.49
SO		3.11	0.21	305.15	304.91
MO		3.26	0.17	293.80	293.82

0		3.50	0.12	319.46	319.29
0.5		3.19	0.20	295.66	295.52

ANOVA, Pr > *F*					

Lipid sources		0.1756	0.0737	0.5845	0.5902
Lysolecithin levels		0.2071	0.0720	0.3237	0.3249
Lipid sources × lysolecithin levels		0.1143	0.0599	0.3864	0.3829

*Note:* Values are means of four replicates. Mean values in the same column with different superscript lowercase letters are significantly different (*p* < 0.05).

#### 3.8.2. Composition of Intestinal Microbiota

The phylum‐level composition and variations of the intestinal microbial community of *M. rosenbergii* are presented in Figure 9. The dominant phyla across all experimental groups primarily included Bacillota, Pseudomonadota, Actinomycetota, and Patescibacteria. The addition of LL significantly altered the relative abundance patterns of these phyla. Specifically, in the FO groups (FO vs. FOLL), LL supplementation reduced the abundance of Bacillota while increasing the relative abundances of Actinomycetota and Patescibacteria. In the SO groups (SO vs. SOLL), LL addition induced a dramatic shift, with Bacillota increasing sharply to become the absolute dominant phylum, whereas Pseudomonadota and Actinomycetota were notably suppressed. Conversely, in the MO groups (MO vs. MOLL), LL supplementation increased the relative abundance of Pseudomonadota but decreased that of Actinomycetota.

**Figure 9:**
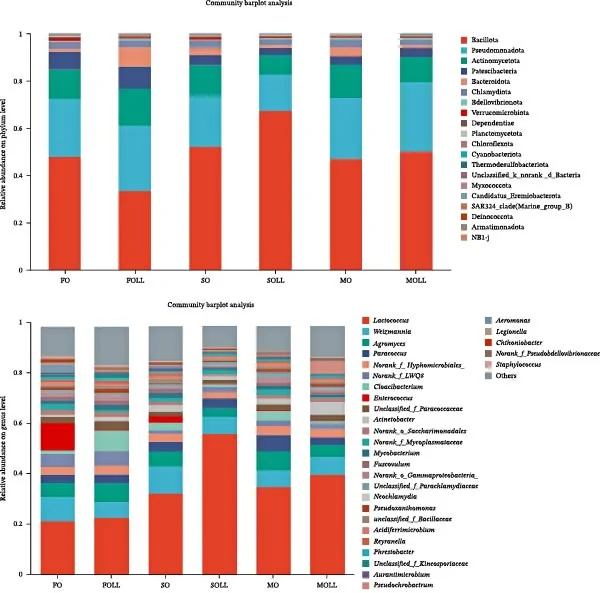
Composition of intestinal bacterial communities at the phylum and genus levels in *M. rosenbergii*.

At the genus level (Figure 9), the grouping showed the main presence of *Lactococcus*, *Weizmannia*, *Agromyces*, and *Paracoccus*. Adding LL drove different changes in particular organism groups, and these changes related to the fat source. In the FO groups, the measure for *Weizmannia* showed decrease following addition of the LL, but *Agromyces* and *Paracoccus* indicated increasing pattern. The SO groups revealed a major change, and addition of the LL produced a large increase in *Lactococcus*, which became the main organism group present, and this occurred with a significant decrease in *Weizmannia*, *Agromyces*, *Paracoccus*, and *Pseudomonas*. In the MO groups, adding LL produced a small increase in Lactococcus and a decreasing pattern for *Weizmannia* and *Agromyces*.

To further elucidate specific microbial responses to LL across lipid sources, a differential analysis was conducted using the Wilcoxon rank‐sum test (Figure 10). In the FO groups (FO vs. FOLL), LL supplementation significantly increased the relative abundance of Pseudaeromonas (*p* < 0.05). In the SO groups (SO vs. SOLL), LL induced a significant restructuring of the microbiota, characterized by a marked enrichment of Lactococcus (*p* < 0.05) and a significant depletion of Mycobacterium, Aeromonas, Pseudomonas, and Desulfovibrio (*p* < 0.05). Finally, in the MO groups (MO vs. MOLL), LL addition significantly up‐regulated TM7a and Pseudaeromonas (*p* < 0.05) while significantly down‐regulating Bradyrhizobium (*p* < 0.05).

**Figure 10:**
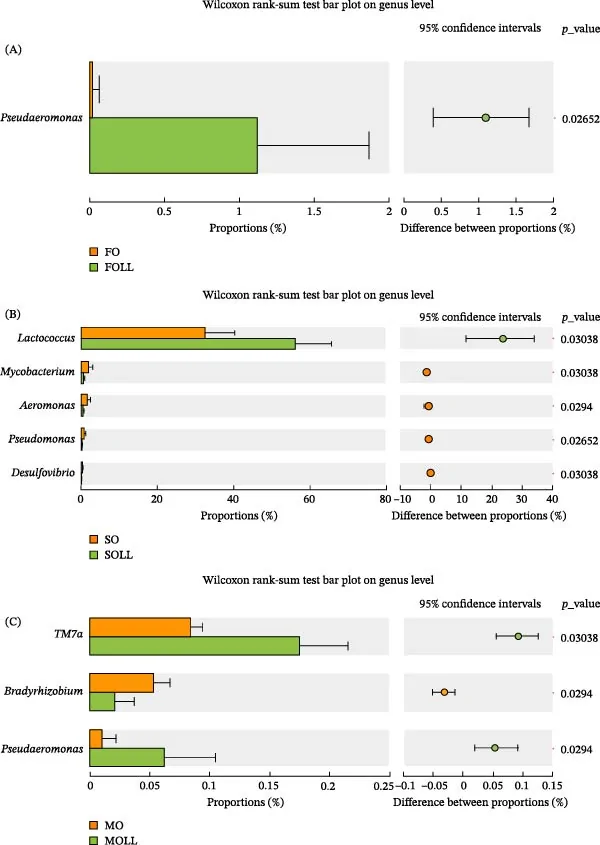
Pairwise comparisons of differentially abundant genera in the intestine of *M. rosenbergii* between lysolecithin‐supplemented and nonsupplemented groups (Wilcoxon rank‐sum test). (A) FO vs. FOLL, (B) SO vs. SOLL, and (C) MO vs. MOLL.

#### 3.8.3. Predicted Functions of Intestinal Microbiota

The heatmap of predicted KEGG functional pathways (level 2) of the intestinal microbiota, based on 16S rRNA sequencing data, is presented in Figure 11. Overall, the functional metabolic profiles of the intestinal microbiota across experimental groups exhibited a high degree of similarity. This suggests that although different lipid sources and LL supplementation altered the taxonomic composition of the community, the core functions of the intestinal microbiota remained relatively stable.

**Figure 11:**
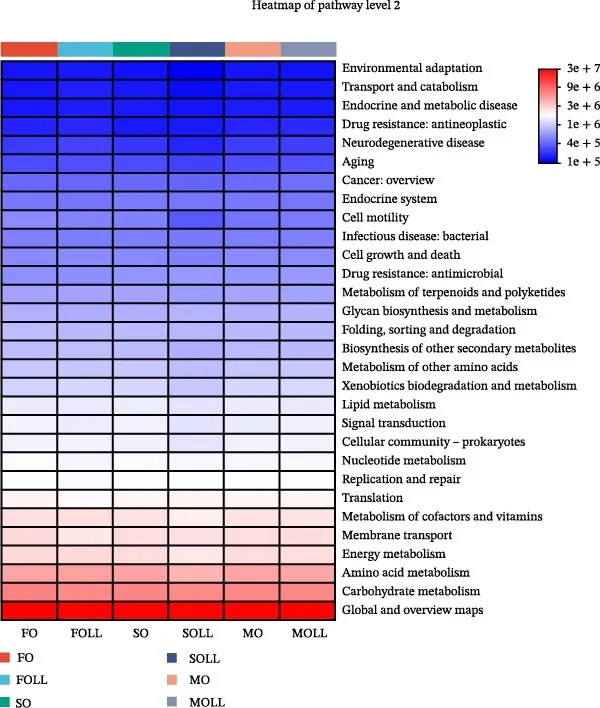
Heatmap of predicted KEGG functional pathways (level 2) of intestinal microbiota in *M. rosenbergii*. The color intensity represents the relative abundance of functional pathways predicted by PICRUSt2. Red indicates higher abundance, while blue indicates lower abundance. Columns represent different experimental groups, and rows represent KEGG level 2 pathways.

In terms of relative abundance, functional genes in the intestine of *M. rosenbergii* were primarily enriched in basal metabolic pathways. Among these, “Global and overview maps” exhibited the highest relative abundance, followed by “Carbohydrate metabolism,” “Amino acid metabolism,” “Energy metabolism,” and “Membrane transport.” Additionally, the pathways for “Metabolism of cofactors and vitamins” and “Translation” also displayed high levels of enrichment.

## 4. Discussion

Growth performance and feed utilization are critical indicators of how aquatic species respond to different dietary lipid sources [44]. In this study, both lipid source and LL supplementation significantly affected these parameters in *M. rosenbergii*. Prawns fed the MO diet (1:1 FO–SO) achieved significantly higher final body weight, WG, and SGR than those fed single lipid sources. The improved FCR observed with both the mixed‐oil diet and LL supplementation indicates enhanced nutrient utilization efficiency. Similar improvements in growth and feed use have been reported in prawns and fish fed balanced lipid mixtures, where marine and vegetable oils complemented each other’s fatty acid profiles [45]. A significant interaction between the lipid source and LL was observed, indicating that emulsifier supplementation modulates lipid utilization efficiency. Supplementation with 0.5% LL improved the growth performance and feed utilization in all dietary lipid treatments. The group receiving the FO–SO blend with LL exhibited the highest growth performance and optimal feed efficiency. This group performs significantly better than groups receiving FO, SO, and MO without the addition of LL. Similar trends were reported by Qiu et al. [38], who found that LL supplementation enhanced growth, AOC, and lipid metabolism in *M. rosenbergii* through improved emulsification and absorption of dietary lipids. Likewise, Qiu et al. demonstrated that a lecithin‐enriched diet further promoted growth, lipid metabolic efficiency, and disease resistance in *M. rosenbergii*, confirming the broader role of phospholipid additives in optimizing energy utilization [42].The current study also demonstrates that optimizing dietary lipid formulation in combination with emulsifying additives such as LL effectively enhances lipid utilization and unlocks the growth potential of cultured aquatic species. The reduction in growth that appears in the SO dietary treatment likely occurs due to deficiency of long‐chain n‐3 PUFAs. SO contains no EPA and DHA, and *M. rosenbergii* has limited enzymatic capacity for biosynthesis of long‐chain PUFAs [46, 47]. The MO diet, through the inclusion of FO, supplied essential n‐3 LC‐PUFA and produced a more balanced dietary n‐3/n‐6 ratio. This formulation allowed the diet to meet the physiological requirements of *M. rosenbergii* while also potentially reducing feed costs by partially replacing FO with SO. The growth‐promoting effect of LL observed in this study is mainly attributed to its ability to enhance lipid bioavailability. Crustaceans lack bile salts and show weak natural emulsification capabilities. LL, a lysophospholipid characterized by a high HLB (HLB), acts as an efficient emulsifier that lowers the oil and water interfacial tension. This reduction in interfacial tension facilitates lipid emulsification and promotes the formation of stable mixed micelles, thereby enhancing lipid digestion and absorption [48, 49].

The composition of the whole prawn shows efficiency in depositing nutrients and the current state of processes in the organism. In this work, the interaction between the source of lipids and LL did not show significant effects on moisture, protein, and ash but showed significant effects on lipid levels in the whole organism. The group receiving mixed oil (MO) showed the highest deposition of lipids (3.01%), and this measure was significantly higher than the group receiving FO (2.38%). LL provided as a supplement reduced the accumulation of lipids, and the measure decreased from 2.84% to 2.57%. This finding supports the observations of Qiu et al. [42] and Chen et al. [34], who reported that supplementation with exogenous phospholipids can mitigate excessive lipid deposition commonly observed when plant‐based oils are used in aquafeeds.The process by which LL operates involves its function as a substance that promotes the formation of small droplets, and this occurs because it has a high HLB measure. By promoting the formation of small lipid droplets and the transport of structures combining lipids and proteins, it increases the use of lipids through the process of beta‐oxidation rather than promoting deposition. The diet using MO provided a distribution of fatty acids showing different types in similar amounts, but providing LL in addition to this further improved the movement of substances through processes to control lipids in the organism. These results support the idea that LL reduces deposition of lipids in *M. rosenbergii* by improving the processes that use lipids, and this provides a foundation in theory for increased efficiency in aquatic animals.

The composition of fatty acids in the tissue generally reflects the dietary fatty acid profiles [50–52]. In the present study, lipid sources remained the primary determinant of the fatty acid composition. The diet using FO significantly increased n‐3 PUFA, particularly EPA and DHA, in both tissues. The diet using SO produced the highest content of n‐6 PUFA, mainly linoleic acid, while the MO group exhibited an intermediate profile between these two. This supports previous findings in *Larimichthys crocea*, where replacing FO with SO significantly reduced n‐3 PUFA and increased n‐6 PUFA [53]. However, LL supplementation exerted significant tissue‐specific modulation on lipid metabolism. In muscle tissue, LL supplementation significantly reduced the accumulation of ΣMUFA and Σn‐6 PUFA independent of lipid sources. This reduction aligns with the upregulated expression of lipid oxidation genes observed in the hepatopancreas, suggesting that LL promotes the mobilization and β‐oxidation of fatty acids for energy rather than their storage in muscle. More importantly, a significant interaction between lipid sources and LL was observed in the hepatopancreas. Specifically, LL supplementation decreased hepatopancreatic Σn‐3 PUFA in the FO and SO groups, whereas an opposite response was observed in the MO group, where Σn‐3 PUFA increased following LL supplementation. This contrasting pattern indicates that the effect of LL on fatty acid utilization and retention is dependent on the dietary lipid background. In the FO group, the reduction in Σn‐3 PUFA may be partly associated with enhanced fatty acid utilization, as LL markedly upregulated *Pparα*, *FABP*, *CPT1*, and *ACOX1*, indicating increased fatty acid transport and β‐oxidation. In contrast, the increased Σn‐3 PUFA in the MO‐LL group may reflect greater retention or redistribution of n‐3 fatty acids under the more balanced fatty acid supply provided by the MO diet. These findings further demonstrate that LL modulates lipid metabolism in a lipid‐source‐dependent manner.

Hemolymph biochemical parameters are key biomarkers for evaluating crustacean health and metabolic status. Current results indicated that hemolymph cholesterol levels in the MO group were significantly higher than those in the FO and SO groups. However, dietary LL supplementation significantly reduced cholesterol levels in both FO and SO groups. Regarding TG, the SO group exhibited the highest levels, which significantly decreased following LL supplementation. Conversely, TG levels in the MO group were generally elevated and remained unaffected by LL supplementation. These variations may be attributed to differences in the dietary fatty acid composition and phospholipid metabolism. Zhu et al. [54] reported that dietary LL supplementation in *Acanthopagrus schlegelii* reduced circulating lipids and alleviated lipid metabolism damage induced by a high‐fat diet. In the present study, elevated TG levels in the SO group might be linked to excessive lipid deposition, driven by the high ω‐6 fatty acid content in SO. Acting as an emulsifier, LL promotes lipid digestion, absorption, and utilization, thereby effectively lowering hemolymph lipid concentrations. Overall, hemolymph biochemical analysis confirmed the positive role of LL in regulating lipid metabolism and improving lipid profiles, physiologically verifying the feasibility of enhancing the lipid utilization efficiency.

AOC and digestive enzyme activity are critical biomarkers reflecting the oxidative stress response and nutrient digestion efficiency in crustaceans [39, 55]. The results indicated that hepatopancreatic T‐AOC and SOD activity in the FO and MO groups were significantly higher than those in the SO group, with the FO group exhibiting the highest levels. Furthermore, LPS activity in the FO group was significantly higher than that in the SO group. Notably, dietary LL supplementation significantly increased SOD activity and reduced MDA content. However, T‐AOC and LPS activity remained unaffected by LL supplementation. These findings suggest that both FO substitution and LL supplementation effectively enhanced the antioxidant defense capacity of *M. rosenbergii*. Consistent with these results, previous studies on hybrid grouper (*Epinephelus fuscoguttatus ♀* × *E. lanceolatus ♂*) [56], Pacific white shrimp (*Litopenaeus vannamei*) [57], Chinese mitten crab (*Eriocheir sinensis*) [48], and crayfish (*Procambarus clarkii*) [58] demonstrated that dietary lecithin supplementation enhanced antioxidant enzyme activity and mitigated oxidative damage. FO is rich in ω‐3 PUFAs and natural antioxidants, which contribute to maintaining cell membrane stability and mitigating oxidative damage [59]. To further elucidate the molecular mechanism underlying these enzymatic changes, we analyzed the expression of *Nrf2*, a key transcription factor regulating antioxidant defense. Our results showed that *Nrf2* expression in the FO‐LL group was up‐regulated by nearly 3‐fold compared to the unsupplemented group. In conclusion, the analysis of antioxidant parameters and digestive enzyme activity confirmed that appropriate FO and LL inclusion enhanced the antioxidant defense system and lipid digestion capacity of *M. rosenbergii*.

Histological assessment of the hepatopancreas serves as a direct method to evaluate the impact of feed on the health of this primary digestive organ in crustaceans [60]. H&E staining results indicated that hepatopancreatic tubules in the FO and MO groups exhibited regular structures with tightly arranged cells and negligible vacuolization. In contrast, the SO group exhibited slight cell vacuolation and loosely organized lumens. Dietary LL supplementation improved hepatopancreatic morphology across all groups. Notably, lipid vacuoles in the SO‐LL group were significantly reduced, while the MO‐LL group displayed the most compact and intact tissue structure. Quantitative analysis of oil red O staining further revealed that in the absence of LL (0%), the lipid droplet area in the SO group was the highest, followed by the FO group and the MO group (lowest). Supplementation with 0.5% LL significantly reduced lipid droplet deposition in the FO and SO groups, but had no significant effect on the MO group. These findings align with Lin et al. [61] in *Eriocheir sinensis*, demonstrating that phospholipid supplementation mitigates lipid droplet accumulation in the hepatopancreas and protects tissue structural integrity. Elevated lipid deposition in the SO group may be attributed to the high linoleic acid (n‐6 PUFA) content in SO, which potentially promotes hepatopancreatic lipid deposition and oxidative stress. Conversely, the more balanced fatty acid profiles in the FO and MO groups mitigated lipid accumulation. Furthermore, LL, acting as an emulsifier, promotes lipid transport and metabolism, facilitating the catabolism or clearance of excess lipids, thereby effectively alleviating hepatopancreatic lipid deposition [62]. In conclusion, selecting a suitable oil source combined with LL supplementation can effectively maintain hepatopancreatic health.

The expression level of genes related to lipid metabolism reveals the molecular mechanism of the above physiological changes [63]. *SREBP* showed a high baseline expression in MO group. However, the addition of LL significantly decreased the expression of *SREBP* in FO group, suggesting that LL may inhibit lipid synthesis under FO feeding conditions. *Pparα* was significantly up‐regulated in FO‐LL group, and the expression level was the highest in all groups, indicating that LL activated the lipid *β*‐oxidation pathway under FO condition. Similarly, downstream oxidation‐related genes such as *FABP*, *CPT1*, and *ACOX1* were significantly up‐regulated in FO‐LL group. On the contrary, *ACC* and FAS, the key enzymes of fatty acid synthesis, were highly expressed in SO group. Notably, LL supplementation significantly increased the expression of *FAS* and *DGAT* in SO group, which may further aggravate the lipid deposition in this group. Overall, the above results show that the role of LL is feed‐dependent, that is, it mainly enhances fatty acid oxidation and inhibits synthesis under the background of FO, while it may promote synthesis and storage pathways under the background of SO. These findings are consistent with the research results of Limwachirakhom et al. [27], that is, LL can increase the expression of oxidation genes, thus revealing the potential molecular mechanism of LL improving the lipid metabolism of crustaceans. To sum up, gene expression analysis confirmed the role of LL in promoting lipid utilization and regulating metabolic flux, which provided molecular evidence for the conclusion of optimizing the energy metabolism in this study.

The organisms that live in the intestinal system provide a second collection of genetic material for the organism, and these organisms function to obtain nutrients and provide defense through interactions between the organism and the microbes [64]. The findings show that the interaction between sources of dietary lipid and LL did not produce significant changes in the diversity in the intestinal organisms. Studies in mammals show that LLs that allow mixing often produce disruption in the microbial community, but these findings suggest that crustaceans maintain a stable core group of microbes [65, 66]. The SO‐LL group showed a specific increase in Lactococcus. SO contains high levels of linoleic acid, and the properties of LL that allow mixing may increase the availability of fatty acids that derive from plants, and this provides conditions that support bacteria producing lactic acid that can process fatty acids [67, 68]. Lactococcus functions as a beneficial organism and can increase defense in the organism and prevent pathogen establishment through competition for space [69]. This increase may indicate a response that occurs when vegetable oil combines with LL that allows mixing, and this may reduce stress in the intestinal system that occurs from diets using plants. The FO and MO groups showed that LL maintained high levels of Agromyces and Pseudaeromonas. This suggests that LL supports microbes that function in protein breakdown and processing of complex lipids in the context of FO and mixed diets. Although LL supplementation induced distinct taxonomic shifts, particularly in the SO group, PICRUSt2 analysis showed that the predicted KEGG functional profiles remained largely similar among treatments. This suggests that the intestinal microbiota maintained a relatively stable core functional capacity despite changes in community composition, possibly reflecting functional redundancy among bacterial taxa. Nevertheless, the enrichment of *Lactococcus* and the reduced abundance of Aeromonas, Pseudomonas, and Desulfovibrio in the SO‐LL group may indicate changes in specific host–microbe interactions that are not captured by broad KEGG Level 2 categories. Therefore, these taxonomic changes may have biological relevance, but their direct contribution to the physiological responses of *M. rosenbergii* cannot be established from the present 16S rRNA‐based functional predictions. Further metagenomic or metabolomic analyses would be required to confirm their functional significance.

## 5. Conclusion

In conclusion, dietary lipid source and LL supplementation significantly and interactively affected growth performance, lipid utilization, hepatopancreatic health, and gene expression related to lipid metabolism in *Macrobrachium rosenbergii*. A FO–SO blend (1:1, *w/w*) supplementation with 0.5% LL enhanced growth and feed efficiency, producing the best overall performance. LL improved lipid utilization and AOC, alleviated SO–induced lipid accumulation and hepatopancreatic damage, and modulated the expression of key genes involved in lipid transport, fatty acid oxidation, lipid synthesis, and oxidative stress responses, with effects dependent on lipid source. These findings demonstrate that combining a FO–SO blend with LL is an effective strategy to improve performance and metabolic health while reducing reliance on FO in freshwater prawn diets.

## Funding

This study was supported by the xuqiyou (Grant 2023C02024).

## Conflicts of Interest

The authors declare no conflicts of interest.

## Data Availability

The data are available upon request from the authors.
